# Practice of code of ethics and associated factors among medical doctors in Addis Ababa, Ethiopia

**DOI:** 10.1371/journal.pone.0201020

**Published:** 2018-08-08

**Authors:** Mesafint Abeje Tiruneh, Birhanu Teshome Ayele

**Affiliations:** 1 Ethiopian Food, Medicine and Healthcare Administration and Control Authority, Addis Ababa, Ethiopia; 2 Department of Statistics, Addis Ababa University, Addis Ababa, Ethiopia; Emory University, School of Public Health, UNITED STATES

## Abstract

**Introduction:**

In the health sector, questions are being raised about the possible threats to the accepted principles of ethics such as autonomy, beneficence, non malfeasance and justice in the delivery of health care. There is limited information in Ethiopia regarding to practice of code of ethics among medical doctors. Hence, this study aimed to assess practice of code of ethics and associated factors among medical doctors working in governmental and private hospitals in Addis Ababa, Ethiopia.

**Methods:**

Institution based cross sectional quantitative study triangulated with qualitative study was conducted among 500 medical doctors working in governmental and private hospitals and three key informants from Federal Ministry of Health, Ethiopian Food, Medicine and Healthcare Administration and Control Authority and Ethiopian Medical Association in Addis Ababa from May 8, 2017 to June 30, 2017. Data were collected using pretested self-administered structured questionnaire and semi-structured questionnaire. Binary Logistic Regression and Content Analysis methods were used for the quantitative and qualitative data analysis respectively.

**Results:**

The study showed that only 152 (30.4%) of medical doctors had good practice of code of ethics. The odds of having good practice of code of ethics among medical doctors in the age group of 25–29 years were 2.749 times the odds of those in the age group of 30–34 years (AOR = 2.749, 95% CI: 1.483, 5.096), medical doctors working in governmental hospitals were 65.4% less likely to have good practice of code of ethics compared to those working in private hospitals (AOR = 0.346, 95% CI: 0.184, 0.652), knowledgeable medical doctors were 83.5% more likely to have good practice of code of ethics compared to those who were not knowledgeable about code of ethics (AOR = 1.835, 95% CI: 0.999, 3.368), and the odds of having good practice of code of ethics among medical doctors with favourable attitude were 7.404 times the odds of those with unfavourable attitude towards code of ethics (AOR = 7.404, 95% CI: 4.254, 12.887). Furthermore lack of motivation, unfavorable working environment, working at various health facilities simultaneously, public awareness, taking courses on medical ethics, lack of unethical conduct reporting and complaint handling system, incompetence of medical doctors, and weak collaboration among key stakeholders were identified as determinants of practice of code of ethics.

**Conclusions:**

Only 30.4% of medical doctors had good practice of code of ethics. This indicates that practice of code of ethics among medical doctors in Addis Ababa is poor. The factors associated with practice of code of ethics were age, type of hospital, knowledge, attitude, lack of motivation, unfavorable working environment, working at various health facilities simultaneously, public awareness, medical ethics course, lack of unethical conduct reporting and compliant handling system, incompetence of medical doctors and weak collaboration among key stakeholders. Hence, awareness creation and attitudinal change on code of ethics by continuous training, implementation of integrated medical ethics course, enforcement of code of ethics and continuing professional development (CPD) implementation are important.

## Introduction

Ethics is the moral principle which attempts to verify what is morally right and what is morally wrong in human action and also has been described as the science of morals and rules of conduct recognised in human life [1–3]. Code of ethics in medical practice describes what is expected from medical doctors registered and licensed to practice medicine. It determines the principles that characterise good medical practice and clarify the standards of professional ethics expected from medical doctors by their professional peers, other health professionals and the community [4].

Even though professional ethics as applied to practice of medicine years back to the ancient civilization by the symbolic adherence to the Hippocratic Oath, codes of ethics and laws regulating the profession have been developed and updated from time to time based on contexts [5]. Although medical ethics principles are universally accepted by various countries, each country can make certain modifications and formulate specific interpretations consistent with the existing culture, religious beliefs, social and norms, laws of the land, and standards of medical practice in the health system [6].

In Ethiopia, based on Food, Medicine and Healthcare Administration and Control Proclamation No.661/2009, Health Professionals’ Code of Ethics has been developed and endorsed through Regulation No. 299/2013 to assure health professionals ethics for the safety of clients and patients. According to this regulation, Federal Health Professionals Ethics Committee (FHPEC) was reorganized in 2014 to examine, investigate and propose appropriate administrative measures to the Ethiopian Food, Medicine and Healthcare Administration and Control Authority (EFMHACA) on complaints made with respect to substandard health services, incompetent and unethical health professionals [7].

Violation of code of ethics in clinical practice leads to disability and death of clients and patients. Hence, administrative measures have been taken on health professionals including medical doctors who violate code of ethics [8]. In the health sector, questions are being asked about the possible threats to the accepted principles of ethics such as autonomy, beneficence, non malfeasance and justice in the delivery of health care [9]. There has been growing public concern regarding the ethical conduct of medical doctors. The role of ethics has become a moral, legal and basic need in almost all stages of medical practice [1].

Not practicing code of ethics, poor management and solution for medical ethics cases not only threaten to weaken patient-doctor relationship, but may also lead to low quality service provision and potentially high incidences of violence and abuse [10, 11]. Although medical doctors are supposed to provide comprehensive health care, patients’ dissatisfaction on the health care they provide is on the rise. Dissatisfaction is reflected in expressions about poor ethical conduct among others [12]. With impressive advances in medical sciences, many ethical issues related to healthcare have risen which needs to be dealt with extreme professionalism in line with various codes of ethics [2]. However, despite all codes and regulations, reports of unethical behaviour of medical doctors are common [1, 2].

Nowadays unethical medical practice is a serious issue in the world. The effects of unethical medical practice are wide reaching and harming many clients/patients who come to hospitals in search of compassionate medical services [13]. The increase in litigation against medical doctors is an immediate and hot issue [14, 15].

A cross-sectional study conducted on medical and dental professionals of Jaipur city, Rajasthan indicated that practice scores of medical doctors varied with their work experience and the difference in the scores was highly significant. Best practice was found among those with the work experience from 10–20 years. Medical doctors working in governmental health facilities had good practice of code of ethics than those working in private health facilities. This was due to the fact that medical doctors in governmental health facilities work under ethics committee that supervises them to work according to the ethical principles. However, the mean practice score difference of medical doctors had no significant association with level of education and age [1]. The result of a cross-sectional descriptive study conducted in Lahore, Pakistan among medical doctors in a tertiary care teaching hospital indicated that medical doctors had poor practice of Pakistan Medical and Dental Council code of ethics [16]. Another study conducted through self-administered structured questionnaire in Nigeria identified gaps in practice of code of ethics among medical doctors [17].

For medical doctors to practice medical ethics, their clinical expertise and subject matter training need to be honed by appropriate medical ethics training [18]. Therefore, medical doctors are expected to know ethical principles and apply them in their clinical practice [19]. Ethics teaching has been shown profound influence on medical professionals' attitudes and practice [20]. It should be part of ongoing medical education. Effective medical ethics education improves the goals of medicine in tangible ways [21]. It is important to identify deficiencies of medical doctors on ethical issues and arrange sensitization and appropriate training [12, 22].

To assure health professionals ethics in Ethiopia, health professionals’ Code of Ethics has been developed and endorsed through Regulation No. 299/2013 by the Council of Ministers in 2013. Health regulatory bodies expect all medical doctors to respect all articles/statements included in the code of ethics [7]. Even though regulations have been endorsed and the Federal Health Professionals Ethics Committee has been established in Ethiopia, clients and patients have complaints on health professionals’ ethics especially on medical doctors. The Federal Health Professionals Ethics Committee has been examining complaints of clients/patients related to violation of code of ethics since 2002. A review of the three-year (from January 2011 to December 2013) report of the Federal Health Professionals Ethics Committee was done in December 2014. From a total of 60 complaints submitted to the committee, 51 (85%) were against medical doctors. Among the total cases/complaints, 14 cases had ethical breach and/or negligence/incompetence. Administrative measures were taken on these involved accordingly [8]. From January 2014 to December 2016 a total of 65 complaints (cases) related to unethical health professionals practice were submitted to the Federal Health Professionals Ethics Committee of which 38 cases (58.5%) were on medical doctors [23].

There is limited information regarding practice of code of ethics while medical doctors provide medical service and associated factors in Ethiopia in general and in Addis Ababa in particular. Since Ethiopia has its own culture, education, health care, and regulatory system, it was necessary to conduct a research in the country’s context. The findings of this study will provide information about practice of code of ethics and associated factors among medical doctors for concerned stakeholders to develop strategies and to take appropriate measures to narrow the gaps and ensure the health of the public.

Hence, this study aimed to assess practice of code of ethics and associated factors among medical doctors working in governmental and private hospitals in Addis Ababa, Ethiopia.

## Methods

### Study design and setting

Institution based cross-sectional quantitative study triangulated with qualitative study was conducted to assess practice of code of ethics and associated factors among medical doctors working in governmental and private hospitals in Addis Ababa from May 8, 2017 to June 30, 2017. Sequential explanatory mixed method design strategy was used to triangulate the findings of the quantitative study with the findings of the qualitative study. Addis Ababa, the capital city of Ethiopia has ten sub-cities and a total population of 3,273,001 [24]. During the study period, there were 12 governmental hospitals and 26 private hospitals in Addis Ababa. There are three types of hospitals; primary hospital, general hospital and comprehensive specialized hospital. The standards/requirements are the same for governmental and private hospitals [25–27]. Hospitals are regulated by EFMHACA and Addis Ababa Food, Medicine and Healthcare Administration and Control Authority (AAFMHACA) based on their level. Medical doctors are registered, licensed and regulated by EFMHACA [28]. According to the data collected from EFMHACA, AAFMHACA and hospitals there were a total of 1,804 medical doctors working in governmental hospitals (1,477) and private hospitals (327) in Addis Ababa.

### Study population

The study participants were medical doctors working in selected governmental and private hospitals in Addis Ababa with a minimum of six months work experience.

### Sample size and sampling procedure

The sample size for the quantitative study was determined using single population proportion formula by considering the following statistical assumptions: 95% confidence interval (CI), 50% proportion (as there were no similar studies to be taken for proportion), 5% marginal error, design effect of 1.5 for multi-stage sampling and a 10% non-response rate. Hence, the final sample size for this study was 524.

Multi-stage sampling technique was used to select the study participants for the quantitative study. In Addis Ababa, there were a total of 1,804 medical doctors (1,477 medical doctors in 12 governmental hospitals and 327 medical doctors in 26 private hospitals). Six governmental hospitals and eight private hospitals were selected by simple random sampling method. Then, by proportional allocation 429 medical doctors from six governmental hospitals and 95 medical doctors from eight private hospitals were selected using simple random sampling technique.

For the qualitative study the sample size was determined in advance to select key informants from the relevant organizations/stakeholders: FMoH, EFMHACA and EMA. One key informant from each organization was selected. Hence, the total sample size for the key informant interview was three. Purposive sampling technique was used to select a key informant from each organization. The key informants were selected based on their experience and responsibility in the respective organizations. In addition, they have first hand information about Ethiopia’s health professionals’ code of ethics.

### Data collection tools, procedures and quality assurance

The quantitative data were collected using self-administered structured questionnaire developed by the investigators based on Ethiopia’s health professionals’ code of ethics and literature review of related studies. Before the actual data collection, the self-administered structured questionnaire was pretested for its completeness on five percent randomly selected medical doctors from governmental and private hospitals that were not included in the study and necessary amendments were made.

To assess knowledge of code of ethics among medical doctors 16 statements of code of ethics which are directly related with medical practice were used with multiple choice questions. Each statement was coded, computed and the score was dichotomized in to knowledgeable (participants who scored ≥ 75% on knowledge based questions) and not knowledgeable (participants who scored < 75% on knowledge based questions).

Medical doctors’ attitude and practice regarding code of ethics were assessed using 16 attitude and 16 practice based questions directly related to medical practice and scored with five Likert scales (0–4). Then, all attitude and practice based questions were coded, computed and the scores were categorized in to favorable attitude (participants who scored ≥ 75% on attitude based questions) and unfavorable attitude (participants who scored <75% on attitude based questions), and good practice (participants who scored ≥ 75% on practice based questions) and poor practice (participants who scored < 75% on practice based questions) respectively. The collected data were checked for consistency and completeness before analysis. Finally, Epi-Info version 7.2.1.0 was used to control and manage errors resulting from data entry process.

The key informant interviews were conducted face to face by the principal investigator (corresponding author of this study) using semi-structured open-ended interview questionnaire with probing questions. The interviews were tape recorded and notes were taken properly.

### Data management and analysis

The collected quantitative data were coded and entered into Epi-Info version 7.2.1.0 and exported to SPSS version 23.0 software for analysis. Participants’ socio-demographic characteristics, knowledge, attitude and practice were described using the relevant descriptive statistics. Univariate analysis was done at 25% level of significance to screen out potentially significant independent variables. The association between the dependent and independent variables were analyzed using Binary Logistic regression model. The adequacy of the final multiple Binary Logistic regression model was checked using the Hosmer and Lemeshow goodness of fit test and the final model fitted the data well (p-value = 0.096). For the Binary Logistic regression model, 95% confidence interval for odds ratio was constructed and variables with p-value ≤ 0.05 were considered as statistically significant.

For the qualitative study the tape recorded audios and notes of interviews were transcribed using non-verbatim transcription technique. Two experienced reviewers read the transcript and gave comments for the content analysis before data synthesis and report writing. The transcribed scripts were intensively read to identify key themes and the data were synthesized thematically. The data were analyzed manually and Content analysis method was used.

### Ethics approval and consent to participate

The study was approved by GAMBY Medical and Business College (GAMBY, IRERC, 2017), Addis Ababa Health Bureau (Ref. No: AAHB/6287/227) and St. Paul Specialized Hospital Millennium Medical College (Ref. No: PM 23/216) Research Ethics Review committees. Permission and support letter were obtained from Addis Ababa Health Bureau. Prior to the data collection, permission was obtained from all hospitals and organizations selected for this study. Written consent was taken from the study participants and key informants after briefing them the objective of the study. To ensure confidentiality, personal identifiers like name were not registered in the data collection tool; finally the collected data were kept and locked after completed data entry.

## Results

### Socio-demographic characteristics

Among 524 medical doctors, 500 responded that makes the response rate 95.4%. Majority, 306 (61.2%) of the study participants were males and half of the study participants were in the age group of 25–30 years. Three hundred seventeen (63.4%) of the study participants were Orthodox Tewahido Christians. Regarding level of education, 320 (64%) of the respondents were general practitioners. Two hundred thirty (46%) of the respondents had less than four years of work experience, and the median monthly income of study participants was 10,000.00 Ethiopian Birr (ETB) (IQR 8,000.00–10,470.00). Two hundred thirty nine (47.8%) of study participants were satisfied with their work (**Table 1**).

**Table 1:** Socio-demographic characteristics of medical doctors in Addis Ababa, 2017 (n = 500).

Variables	Frequency	Percent (%)
**Sex**		
Male	306	61.2
Female	194	38.8
**Age in years**		
25–29	251	50.2
30–34	147	29.4
>34	102	20.4
**Religion**		
Orthodox Tewahido	317	63.4
Protestant	84	16.8
Muslim	55	11.0
Catholic	23	4.6
Others*	21	4.2
**Level of education**		
General Practitioner	320	64.0
Specialist	180	36.0
**Work experience in years**		
<4	230	46.0
4–7.9	184	36.8
≥8	86	17.2
**Level of satisfaction on work**		
Very satisfied	76	15.2
Satisfied	239	47.8
Unsure	71	14.2
Dissatisfied	87	17.4
Very dissatisfied	27	5.4

Others*: include Jehovah Witness, Wakefeta, and unspecified religions

### Institutional factors

Four hundred twenty three (84.6%) of the study participants were from governmental hospitals. From the total study participants, 459 (91.8%) took medical ethics course which has its own curriculum during their medical education and those who took medical ethics course, the median year since they took the course was six years (IQR: 5–8). Majority, 417 (83.4%) of the respondents have not taken any training on code of ethics/ medical ethics after medical school/since qualification and those who took training on code of ethics/ medical ethics, the median year since they took the training was two years (IQR: 1–4) (**Table 2**).

**Table 2:** Institutional factors for practice of code of ethics among medical doctors in Addis Ababa, 2017 (n = 500).

Variables	Frequency	Percent (%)
**Type of hospital**		
Governmental	423	84.6
Private	77	15.4
**Took medical ethics course**		
Yes	459	91.8
No	41	8.2
**Had training on code of ethics/ medical ethics since qualification**		
Yes	83	16.6
No	417	83.4

### Knowledge and attitude on code of ethics

From the total study participants, 375 (75%) and 283 (56.6%) of them knew that Ethiopia has Health Professionals Code of Ethics and the existence of Federal Health Professionals Ethics Committee respectively. From those who knew the existence of the Federal Health Professionals Ethics Committee, 186 (65.7%) of them did not know the powers and duties of the Federal Health Professionals Ethics Committee. Three hundred seventy eight (75.6%) of the study participants were knowledgeable about code of ethics, and 303 (60.6%) of the study participants had favorable attitude towards code of ethics (**Table 3**).

**Table 3:** Knowledge and attitude of code of ethics among medical doctors in Addis Ababa, 2017 (n = 500).

Variables	Frequency	Percent (%)
**Knowledge about existence of** **Code of Ethics**		
Yes	375	75.0
No	125	25.0
**Knowledge about existence of FHPEC**		
Yes	283	56.6
No	217	43.4
**Knowledge about powers & duties of FHPEC (n = 283)**		
Yes	97	34.3
No	186	65.7
**Knowledge of code of ethics**		
Knowledgeable	378	75.6
Not knowledgeable	122	24.4
**Attitude of code of ethics**		
Favorable attitude	303	60.6
Unfavorable attitude	197	39.4

### Practice of code of ethics

Among the total study participants, only 152 (30.4%: 95% CI: 26.4, 34.4) of the study participants had good practice and the remaining 348 (69.6%: 95% CI: 65.6, 73.6) of them had poor practice of code of ethics (**Table 4**).

**Table 4:** Responses for practice related questions on of code of ethics among medical doctors in Addis Ababa, 2017 (n = 500).

S.No	Statements of code of ethics	Always n (%)	Very often n (%)	Sometimes n (%)	Rarely n (%)	Never n (%)
1.	How often do you obtain informed consent from a patient before rendering a service?	170 (34.0)	181 (36.2)	101 (20.2)	29 (5.8)	19 (3.8)
2.	How often do you respect patient confidentiality, privacy, choices and dignity?	298 (59.6)	177 (35.4)	21 (4.2)	1 (0.2)	(0.6)
3.	How often do you provide health service for your benefit that does not serve the needs of your patient?	19 (3.8)	26 (5.2)	34 (6.8)	119 (23.8)	302 (60.4)
4.	How often do you work with or give any professional support to other health professional not licensed by appropriate organ?	21 (4.2)	47 (9.4)	110 (22.0)	112 (22.4)	210 (42.0)
5.	How often do you render the same level of care to your clients in over-time and regular practice?	157 (31.4)	171 (34.2)	83 (16.6)	45 (9.0)	44 (8.8)
6.	How often do you provide any preferential treatment to a client/patient by considering the relationship established with you in other health institution where you works?	18 (3.6)	39 (7.8)	145 (29.0)	116 (23.2)	182 36.4)
7.	How often do you use secret remedies to treat a patient?	42 (8.4)	36 (7.2)	49 (9.8)	79 (15.8)	294 (58.8)
8.	How often do you use an apparatus or health technology or intervention which is proved up on investigation to be capable of fulfilling the claims made in regard to it?	154 (30.8)	129 (25.8)	161 (32.2)	34 (6.8)	22 (4.4)
9.	How often do you refuse on ground of your personal belief to provide services such as contraceptive, legal abortion and blood transfusion?	29 (5.8)	57 (11.4)	125 (25.0)	88 (17.6)	201 (40.2)
10.	How often do you sign and write your name on official documents relating to patient care such as laboratory and other diagnostic requests and results, prescriptions, certificates, patient records and other reports?	269 (53.8)	183 (36.6)	38 (7.6)	7 (1.4)	(0.6)
11.	How often do you issue genuine and complete sick leave or certificate of illness?	287 (57.4)	168 (33.6)	37 (7.4)	2 (0.4)	(1.2)
12.	How often do you prescribe medicine or formulations about which you do not know about its composition and pharmacological action?	19 (3.8)	19 (3.8)	61 (12.2)	110 (22.0)	291 (58.2)
13.	How often do you prescribe medicine not registered in the National Medicine List without compelling reason?	8 (1.6)	17 (3.4)	69 (13.8)	133 (26.6)	273 (54.6)
14.	How often do you report impairment in other health professional to the appropriate organ if you are aware of it?	37 (7.4)	46 (9.2)	116 (23.2)	106 (21.2)	195 (39.0)
15.	How often do you report your own impairment to the appropriate organ if you are aware of it?	48 (9.6)	54 (10.8)	105 (21.0)	88 (17.6)	205 (41.0)
16.	How often do you report any unprofessional/unethical conduct of another health professional to the appropriate organ?	59 (11.8)	44 (8.8)	98 (19.6)	105 (21.0)	194 (38.8)

### Factors associated with practice of code of ethics

From univariate analysis of the independent variables age, level of education, type of hospital, level of satisfaction, knowledge of code of ethics and attitude towards code of ethics were significantly associated with practice of code of ethics among medical doctors at 25% level of significance. Sex, age, religion, level of education, type of hospital, work experience, training, level of satisfaction, knowledge of code of ethics and attitude towards code of ethics were considered in the multivariable Binary Logistic regression model. However, only age, type of hospital, knowledge and attitude were found to be significantly associated with practice of code of ethics in the multiple Binary Logistic regression model at 5% level of significance.

Accordingly, the odds of having good practice of code of ethics among medical doctors in the age group of 25–29 years were 2.749 times the odds of those in the age group of 30–34 years (Adjested odds ratio (AOR) = 2.749, 95% Confidence interval (CI): 1.483, 5.096).

Regarding type of hospitals, medical doctors working in governmental hospitals were 65.4% less likely to have good practice of code of ethics compared to those working in private hospitals (AOR = 0.346, 95% CI: 0.184, 0.652).

Knowledgeable medical doctors were 83.5% more likely to have good practice of code of ethics compared to those who were not knowledgeable about code of ethics (AOR = 1.835, 95% CI: 1.001, 3.368).

The odds of having good practice of code of ethics among medical doctors with favourable attitude were 7.404 times the odds of those with unfavourable attitude towards code of ethics (AOR = 7.404, 95%CI: 4.254, 12.887) (**Table 5**).

**Table 5:** Univariate and multivariable analysis for factors associated with practice of code of ethics among medical doctors in Addis Ababa, 2017 (n = 500).

Variables	Practice		COR(95% CI)	AOR(95% CI)	p-value
	Good	Poor			
**Sex**					
Male	96	210	1.127 (0.760, 1.669)	1.481 (0.930, 2.356)	0.098
Female	56	138	1.00	1.00	
**Age in years**					
25–29	91	160	1.965 (1.234, 3.128)	**2.749 (1.483, 5.096)**	0.001*
30–34	33	114	1.00	1.00	
>34	28	74	1.307 (0.730, 2.340)	1.213 (0.471, 3.121)	0.689
**Religion**					
Orthodox	97	220	0.931 (0.555, 1.560)	1.096 (0.610, 1.970)	0.757
Catholic	8	15	1.126 (0.426, 2.978)	3.107 (0.983, 9.817)	0.053
Protestant	27	57	1.00	1.00	
Muslim	13	42	0.653 (0.302, 1.415)	0.581 (0.247, 1.365)	0.213
Others	7	14	1.056 (0.382, 2.916)	1.961 (0.598, 6.431)	0.267
**Level of education**					
General Practitioner	107	213	1.507 (1.001, 2.270)	1.274 (0.725, 2.239)	0.400
Specialist	45	135	1.00	1.00	
**Type of hospital**					
Governmental	121	302	0.595 (0.360, 0.982)	**0.346 (0.184, 0.652)**	0.001*
Private	31	46	1.00	1.00	
**Work experience**					
<4 years	75	155	1.196 (0.785, 1.823)	0.902 (0.523, 1.558)	0.712
4–7.9 years	53	131	1.00	1.00	
> = 8 years	24	62	0.957 (0.542, 1.690)	1.011 (0.380, 2.687)	0.982
**Training**					
No	126	291	1.00	1.00	
Yes	26	57	1.053 (0.633, 1.752)	1.009 (0.563, 1.810)	0.975
**Level of satisfaction**					
Very satisfied	22	54	1.00	1.00	
Satisfied	68	171	0.976 (0.552, 1.726)	0.644 (0.331, 1.252)	0.195
Unsure	19	52	0.897 (0.436, 1.847)	0.744 (0.321, 1.722)	0.490
Dissatisfied	31	56	1.359 (0.701, 2.634)	0.955 (0.444, 2.055)	0.906
Very dissatisfied	12	15	1.964 (0.793, 4.862)	1.902 (0.675, 5.355)	0.224
**Knowledge**					
Knowledgeable	130	248	2.383 (1.434, 3.960)	**1.835 (1.001, 3.368)**	0.050*
Not knowledgeable	22	100	1.00	1.00	
**Attitude**					
Favourable attitude	129	174	5.609 (3.433, 9.163)	**7.404 (4.254, 12.887)**	<0.001*
Unfavourable attitude	23	174	1.00	1.00	

Note:

* Statistically significant at 5% level of significance multivariable analysis

### Qualitative findings

#### Key informants

Three in-depth interviews were done with key informants selected from FMoH, EFMHACA and EMA. The key informants had experience, responsibility and first hand information regarding code of ethics and/or medical ethics in their respective organizations. The first key informant was a medical doctor who used to be a program advisor in medical education and trainer of medical ethics and professionalism in FMoH. The second key informant was a lawyer who was a Director of Medico Legal Directorate and member of the Federal Health Professionals’ Ethics Committee in EFMHACA. The third key informant was a medical doctor who was an Executive Director of EMA with additional responsibility in medical ethics.

#### Practice of code of ethics

The key informant interviews indicated gaps in practice of code of ethics among medical doctors in governmental and private hospitals. One of the key informants said that:

“………we are observing a lot of painful medico legal issues. Patients are crying out loud, because they feel that their rights are violated. ……….medical doctors also defend themselves by saying “no medical doctor is going to his/her hospital just to kill a person.” When we try to balance those issues there are many factors. There is of course a problem in practice of code of ethics like in the other countries developed or developing; there are medical doctors who are unethical”.

Some indicators of the gap in practice of code of ethics were complaints from clients/patients, case submitted to the Federal Health Professionals Ethics Committee and administrative measures taken by EFMHACA such as being assigned under supervision of senior medical doctors for a certain period of time, professional license suspension and revocation on those who violated the code of ethics.

As the key informants stated, the major gaps in practice of code of ethics among medical doctors were lack of information, communication and counseling, lack of respect and consideration to client/patient right and autonomy such as not stating and explaining the consent well for clients/patients and not taking informed consent at all, not availing themselves to provide medical service in their formal working place at assigned working hours, not reporting unprofessional/unethical conducts, negligence, and unethical medical practices as a result of incompetence of medical doctors. One of the key informants complimented that:

“…….patients autonomy is the most violated ethical principle. Because when patients go to operation room for an operation they sign a consent form. But the consent forms are not well explained to the patients. Patients should be told what the problem is, an alternative treatment if any and what is best for them”.

In addition, the other key informant complemented that:

“………some medical doctors do not consider informed consent as a part of medical procedures. This was confirmed by the investigation of the Federal Health Professionals Ethics Committee based on complain of patients”.

#### Knowledge of code of ethics

Key informants indicated knowledge of code of ethics as one of the determinants of practice of code of ethics. Knowledgeable medical doctors could practice code of ethics properly than those not knowledgeable. Even though, there were different mechanisms to aware medical doctors about code of ethics/medical ethics, the finding of this study identified knowledge gap among medical doctors. One of the mechanisms to aware medical doctors is medical ethics course during medical education. Those who graduated from Ethiopian universities before 2004 did not take medical ethics course at all. Two of the key informants stated that the medical ethics course being delivered with two credit hours is not enough to cover the course in detail and an integrated way. The course was also mainly theoretical and not well integrated with the medical practice. In addition, the course did not include the country’s regulation for health professionals’ regulation in general and code of ethics in particular. A new competency based integrated modular curriculum has been developed by FMoH and the pioneer was Medical School of Addis Ababa University. The curriculum was launched in November 2016 to give preventive and regulatory medical ethics course in all medical schools. This was strengthened by one of the key informants that:

“………at the moment because we feel the importance of medical ethics and code of conduct is so big, we are upgrading the education both in preventive and regulatory ethics for the last three years and we are trying to take the model implementing in all medical schools. ……..yet practically I cannot say it is good enough”.

The other mechanism to aware medical doctors is trainings by FMoH during deployment. In addition, based on Compassionate, Respectful and Caring health professionals (CRC) agenda of the Health Sector Transformation Plan (HSTP), CRC Manual which included medical ethics and professionalism has developed and endorsed, and implementation has started. Presentations by EFMHACA on EMA’s and other societies’ annual conferences, and releasing cases and administrative measures taken by EFMHACA to mass-media were among mechanisms to aware medical doctors. In addition, Continuing Professional Development (CPD) system has been launched, and guideline and directive have been developed and distributed. According to the CPD Guideline of the country, it is expected that every medical doctor should take 30 continues education unit (CEU) per year and 150 CEU per five years for renewal of professional license. However, the guideline and directive are not yet implemented in Ethiopia.

Furthermore, EMA has developed and distributed code of ethics and medical ethics manual. In addition, provides trainings, prepares awareness creation sessions on annual conferences, and ethics committee has been established to consult the Executive Committee of EMA. However, the key informants stated that most of the above activities were launched in the past few years and are not enough to narrow the existing knowledge gap.

### Attitude of code of ethics

The key informants revealed that the other determinant of practice of code of ethics was attitude towards code of ethics which differs among medical doctors. This was strengthened by one of the key informants who stated that: *“………attitude is one of the factors that affect the practice of code of ethics that is why FMoH is advocating companionate*, *respectful and carrying approach*.*”*

Medical doctors who have favorable attitude towards code of ethics could practice code of ethics properly than those who have unfavorable attitude. There were some indicators that show unfavorable attitude towards code of ethics among medical doctors such as questioning why a medical doctor who serves many people will be accused, and hiding violation of code of ethics.

#### Working environment and public awareness

One of the key informants indicated that most medical doctors work in various health facilities simultaneously to boost their income. Due to this reason they become exhausted and are not available in their formal work place. This may lead to the violation of code of ethics and substandard medical services. Lack of motivation and unfavorable working environment also contribute to the violation of code of ethics.

In addition to the above determinants, public awareness was influential on practice of code of ethics. Clients/patients who are aware of medical services and their right contribute a lot for good practice of code of ethics by asking their medical doctors about their health status, diagnostic and treatment procedures, risk/adverse effects and benefit of the treatment rendered to them.

#### Enforcement and collaboration

The key informants indicated that there was lack of unprofessional/unethical conduct reporting and medical ethics complaints handling system at hospitals level. According to cases submitted to the Federal Health Professionals Ethics committee in the past three years, violation of code of ethics was relatively the same in governmental and private hospitals. The administrative measures taken by EFMHACA have been mainly based on complaints coming from clients/patients or other bodies.

FMoH is responsible for health care provision and quality of health care, EFMHACA has a mandate for health care regulation including enforcement of code of ethics and EMA is working on medical ethics and professionalism. The three organizations are working in collaboration in different issues including the Federal Health Professionals Ethics Committee, Continuing Professional Development (CPD), and regulation, directive and guideline preparation. However, the collaboration among these stakeholders has been weak in the implementation and enforcement of code of ethics and was not enough to assure practice of code of ethics in medical practice. One of the key informants said that:

“………I do not remember any collaboration between stakeholders in health care regulation including implementation of code of ethics except document preparation such as guideline and policies”.

## Discussion

The main purpose of this study was to assess practice of code of ethics and associated factors among medical doctors working in governmental and private hospitals in Addis Ababa. Our study revealed that only 152 (30.4%) of medical doctors had good practice of code of ethics. This indicates that majority of medical doctors had poor practice of code of ethics. In addition, the qualitative findings of this study indicated gaps in practice of code of ethics among medical doctors. Our finding is in line with studies conducted in Pakistan and Nigeria [16, 17].

Medical doctors who always obtain informed consent from a patient before rendering a medical service were only 170 (34%). Similarly, the qualitative findings indicated lack of information, communication and counseling, lack of respect and consideration to client/patient right and autonomy such as not stating and explaining the consent well for clients/patients and not taking informed consent at all as the major gaps of practice of code of ethics. Only 59 (11.8%) medical doctors always report unprofessional/unethical conduct of other health professionals to the appropriate organ. Similarly, the qualitative finding of this study confirmed not reporting unprofessional/unethical conduct as one of the major gaps. In addition, not availing themselves to provide medical service in their formal working place at assigned working hours, negligence, and unethical medical practices as a result of incompetence of medical doctors were gaps identified in practice of code of ethics.

The findings of this study identified age as one of the factors significantly associated with practice of code of ethics among medical doctors. The odds of having good practice of code of ethics among medical doctors in the age group of 25–29 years were 2.749 times the odds of those in the age group of 30–34 years. This may be due to the fact that young medical doctors are new for medical practice and usually eager to practice properly what they were thought in medical school. However, in a study conducted in Rajasthan age had no significant association with practice of code ethics [1]. The possible explanation for this difference might be commitment and interest of medical doctors for medical practice and/or due to differences in study areas and population characteristics.

Medical doctors working in governmental hospitals were 65.4% less likely to have good practice of code of ethics compared to those working in private hospitals. This may be due to the focus of private hospitals which are mainly profit driven and customer attraction and handling is pivotal for their survival and it may be due to fear of stringent enforcement mechanism of health regulatory bodies. Hence, medical doctors working in private hospitals are expected to be very careful and communicate with their clients/patients properly. Unlikely, a study conducted in Rajasthan indicated that medical doctors working in governmental health facilities had good practice of code of ethics than those in private health facilities. This could be due to the fact that medical doctors in governmental health facilities in Rajasthan work under ethics committee that supervises them to work according to the ethical principles [1].

Knowledgeable medical doctors were 83.5% more likely to have good practice of code of ethics compared to those who were not knowledgeable about code of ethics. According to the qualitative findings, knowledge of code of ethics was also one of the determinants of practice of code of ethics. This may be due to the fact that medical doctors who have knowledge about code of ethics could understand what is right and what is wrong in the medical practice and may practice code of ethics properly.

The odds of having good practice of code of ethics among medical doctors with favourable attitude were 7.404 times the odds of those with unfavourable attitude towards code of ethics. Furthermore, the qualitative study identified attitude as one of the factors associated with of practice of code of ethics. This might be due to the fact that attitude determines what individuals do and favourable attitude prevails individuals to act positively [29].

Moreover, qualitative findings of this study indicated that lack of motivation, unfavourable working environment, working at various health facilities simultaneously, and public awareness were determinants of practice of code of ethics. Lack of motivation and unfavourable working environment may lead medical doctors to be negligent and hide unethical medical practices. Medical doctors who work at various health facilities simultaneously become exhausted and hence might not present in their formal work place at assigned working hours and/or might not have the energy to contribute their utmost. This might contribute to the poor practice of code of ethics. Furthermore, clients/patients who are aware of medical services and their right usually ask medical doctors about their health status, diagnosis and treatment procedures, risk/adverse effects and benefit of treatment/s rendered to them. This may make medical doctors cautious and carful in providing medical service, and this contributes for good practice of code of ethics in medical practice.

Even though, 459 (91.8%) of medical doctors took medical ethics course during medical education, the time (two credit hours) was not enough to deliver the course and integrate with the medical practice. Moreover, the course did not include the country’s Health Professionals’ Code of Ethics. This finding indicates that medical doctors lacked theoretical and practical aspect of code of ethics in medical practice. This might have contributed for the witnessed poor practice of code of ethics among medical doctors.

Furthermore, there is lack of unprofessional/unethical conduct reporting and complaint handling system at hospitals level. This might lead to hide violation of code of ethics in medical practice and increase client/patient suffering. Administrative measures taken by EFMHACA are mainly based on complaints coming from clients/patients or other bodies. This means of regulation may not prevent violation of code of ethics and difficult to identify the root causes of violation of code of ethics in the medical practice. Moreover, the collaboration among key stakeholders has been weak to assure practice of code of ethics in the medical practice. Implementation and enforcement of code of ethics without collaboration could not be effective and contribute to the poor practice of code of ethics.

The poor practice of code of ethics implies weak implementation and enforcement of code of ethics. Non adherence to code of ethics leads to substandard service provision and incidences of violence and abuse [10]. Poor practice of code of ethics results in various consequences such as worsening of the original health condition, failure to treat the original health condition, development of other health problems, unnecessary surgery, increased medical expenses, disability and death [30, 31]. Furthermore, clients/patients might experience psychological distress and unable to trust medical doctors as well as the health care system after unethical medical practice incident [31].

## Conclusion

Practice of code of ethics among medical doctors working in governmental and private hospitals in Addis Ababa was found to be poor. Age, type of hospital, knowledge of code of ethics and attitude towards the code of ethics were significantly associated with practice of code of ethics. Among these significant factors, knowledge and attitude were also determinants of practice of code of ethics according to qualitative findings of this study. The findings of our qualitative study also showed that lack of motivation, unfavorable working environment, working at various health facilities simultaneously, public awareness, medical ethics course, lack of unethical conduct reporting and complaints handling system at hospital level, incompetence of medical doctors, and weak collaboration among key stakeholders as determinants of practice of code of ethics.

Therefore, it is important to aware and change attitude of medical doctors about code of ethics by continuous training, increase public awareness about healthcare delivery and client/patient right, establish institution based health professionals’ ethics committee and unprofessional/unethical conduct reporting and handling system at hospital level, strengthen competency based medical education and well integrated medical ethics course. In addition, enforcement of code of ethics and establish a system that could help to identify the root causes of frequently lodged complaints and grievances related with violation of code of ethics, enforce implementation of Continuing Professional Development (CPD), and strengthen collaboration among key stakeholders are crucial for good practice of code of ethics. We also recommend further researches to be conducted using observational data collection methods and focus group discussions to minimize social desirability response bias which was considered as a limitation of this study.
